# Academic Performance in Adolescent Students: The Role of Parenting Styles and Socio-Demographic Factors – A Cross Sectional Study From Peshawar, Pakistan

**DOI:** 10.3389/fpsyg.2019.02497

**Published:** 2019-11-08

**Authors:** Sarwat Masud, Syed Hamza Mufarrih, Nada Qaisar Qureshi, Fahad Khan, Saad Khan, Muhammad Naseem Khan

**Affiliations:** ^1^Institute of Public Health & Social Sciences, Khyber Medical University, Peshawar, Pakistan; ^2^Department of Medicine, Aga Khan University Hospital, Karachi, Pakistan

**Keywords:** parenting styles, academic performance, adolescent students, Pakistan, care, overprotection, parental bonding instrument

## Abstract

Academic performance is among the several components of academic success. Many factors, including socioeconomic status, student temperament and motivation, peer, and parental support influence academic performance. Our study aims to investigate the determinants of academic performance with emphasis on the role of parental styles in adolescent students in Peshawar, Pakistan. A total of 456 students from 4 public and 4 private schools were interviewed. Academic performance was assessed based on self-reported grades in the latest internal examinations. Parenting styles were assessed through the administration of the Parental Bonding Instrument (PBI). Regression analysis was conducted to assess the influence of socio-demographic factors and parenting styles on academic performance. Factors associated with and differences between “care” and “overprotection” scores of fathers and mothers were analyzed. Higher socio-economic status, father’s education level, and higher care scores were independently associated with better academic performance in adolescent students. Affectionless control was the most common parenting style for fathers and mothers. When adapted by the father, it was also the only parenting style independently improving academic performance. Overall, mean “care” scores were higher for mothers and mean “overprotection” scores were higher for fathers. Parenting workshops and school activities emphasizing the involvement of mothers and fathers in the parenting of adolescent students might have a positive influence on their academic performance. Affectionless control may be associated with improved academics but the emotional and psychosocial effects of this style of parenting need to be investigated before recommendations are made.

## Introduction

Despite residual ambiguity in the term, definitions over time have identified several elements of “academic success” (Kuh et al., 2006; York et al., 2015). Used interchangeably with “student success,” it encompasses academic achievement, attainment of learning objectives, acquisition of desired skills and competencies, satisfaction, persistence, and post-college performance (Kuh et al., 2006; York et al., 2015). Linked to happiness in undergraduate students (Flynn and MacLeod, 2015) and low health risk behavior in adolescents (Hawkins, 1997), a vast amount of literature is available on the determinants of academic success. Studies have shown socioeconomic characteristics (Vacha and McLaughlin, 1992; Ginsburg and Bronstein, 1993; Chow, 2000; McClelland et al., 2000; Tomul and Savasci, 2012), student characteristics including temperament, motivation and resilience (Ginsburg and Bronstein, 1993; Linnenbrink and Pintrich, 2002; Farsides and Woodfield, 2003; Valiente et al., 2007; Beauvais et al., 2014) and peer (Dennis et al., 2005), and parental support (Cutrona et al., 1994; Sanders, 1998; Dennis et al., 2005; Bean et al., 2006) to have a bearing on academic performance in students.

The influence of parenting styles and parental involvement is particularly in focus when assessing determinants of academic success in adolescent children (Shute et al., 2011; Rahimpour et al., 2015; Weis et al., 2016; Checa and Abundis-Gutierrez, 2017; Zhang et al., 2019). The influence may be of significance from infancy through adulthood (Steinberg et al., 1989; Weiss and Schwarz, 1996; Zahedani et al., 2016) and can be appreciated across a range of ethnicities (Desimone, 1999; Battle, 2002; Jeynes, 2007). Previously, the authoritative parenting style has been most frequently associated with better academic performance among adolescent students (Steinberg et al., 1989, 1992; Deslandes et al., 1997, 1998; Aunola et al., 2000; Adeyemo, 2005; Checa et al., 2019), while purely restrictive and negligent styles have shown to have a negative influence on academic performance (Hillstrom, 2009; Parsasirat et al., 2013; Osorio and González-Cámara, 2016). Parenting styles have also been linked to academic performance indirectly through regulation of emotion, self-expression (Deslandes et al., 1997; Weis et al., 2016), and self-esteem (Zakeri and Karimpour, 2011).

Significant efforts have been made to explore and integrate factors which influence parenting stress and behaviors (Belsky, 1984; Abidin, 1992; Östberg and Hagekull, 2000). A number of factors, including parent personality and psychopathology (in terms of extraversion, neuroticism, agreeableness, depression and emotional stability), parenting beliefs, parent-child relationship, marital satisfaction, parenting style of spouse, work stress, child characteristics, education level, and socioeconomic status have been highlighted for their role in determining parenting styles (Belsky, 1984; Simons et al., 1990, 1993; Bluestone and Tamis-LeMonda, 1999; Huver et al., 2010; Smith, 2010; McCabe, 2014). Studies have also highlighted differences between fathers and mothers in how these factors influence them (Simons et al., 1990; Ponnet et al., 2013).

Insight into determinants of academic success and the role of parenting styles can have significant impact on policy recommendations. However, most existing data comes from western cultures where individualistic themes predominate. While some studies highlight differences between the two (Wang and Leichtman, 2000), evidence from eastern collectivist cultures, including Pakistan, is scarce (Masud et al., 2015; Khalid et al., 2018).

The aim of this study is to identify the determinants of academic performance, including the influence of parenting styles, in adolescent students in Peshawar, Pakistan. We also aim to investigate the factors affecting parenting styles and the differences between parenting behaviors of father and mothers.

## Materials and Methods

The manuscript has been reported in concordance with the STROBE checklist (Vandenbroucke et al., 2014).

### Study Design

A cross sectional study was conducted by interviewing school-going students (grades 8, 9, and 10) to assess determinants of academic grades including the influence of parenting styles.

### Setting

The study took place in the city of Peshawar in Pakistan at eight schools, four from the public sector and four from the private sector. The data collection process began in January 2017 concluded in December 2017.

### Study Size

The prevalence of high grades (A and A plus) among adolescent students was between 42.6 and 57.4% in a previous study (Cohen and Rice, 1997 #248). Based on this, a sample size of 376 students was calculated to study the determinants of high grades in adolescent students with a confidence level of 95%. Assuming a non-response rate of approximately 20%, we decided to target 500 students from four public and four private schools. A total of 456 students participated in our study.

### Participants

#### Inclusion Criteria

From the eight schools which provided admin consent to conduct the study, students enrolled in grade 8, 9, or 10 were invited to take part in the study. Following consent from the parents and assent from the student, he or she was included in the study.

#### Exclusion Criteria

Any student unable to understand or fill out the interview *pro forma* or questionnaire independently.

### Data Sources and Measurement

Data was collected through a one on one interaction between each student and the data collector individually. The following tools were used.

#### Demographic *pro forma* (Supplementary Datasheet 1)

A brief and simple *pro forma* was structured to address all demographic related variables needed for the study.

#### Parental Bonding Instrument (PBI) (Supplementary Datasheet 2)

The original version of the Parental Bonding Instrument (Parker et al., 1979), previously validated for internal consistency, convergent validity, satisfactory construct, and independence from mood effects in several different populations, including Turkish and Chinese (Parker et al., 1979; Parker, 1983, 1990; Cavedo and Parker, 1994; Dudley and Wisbey, 2000; Wilhelm et al., 2005; Murphy et al., 2010; Liu et al., 2011; Behzadi and Parker, 2015), was employed in our study. This tool, composed of 25 questions, assesses parenting styles as two independent measures of “care” and “control” as perceived by the child. It is filled out separately for the father and the mother. It is available online for use without copyright. The use of PBI has been validated for British Pakistanis (Mujtaba and Furnham, 2001) and Pakistani women (Qadir et al., 2005). A paper by Qadir et al. on the validity of PBI for Pakistani women, reports the Cronbach alpha scores to be 0.91 and 0.80 for the “care” and “overprotection” scales, respectively (Qadir et al., 2005).

The demographic *pro forma* and the parental bonding index were translated into Urdu by an individual fluent in both languages and validated with the help of an epidemiologist and two experts in the field (Supplementary Datasheet 3). Pilot testing of translated versions was done with 20 students to ensure clarity and assess understanding and comprehension by the students. Both versions for the two tools were provided in hard copy to each student to fill out whichever one he/she preferred. The data collector first verbally explained the items on the demographic *pro forma* and the PBI to the student following which the student was allowed to fill it out independently.

### Variables

Using the data sources mentioned above, data was collected for the following variables.

#### Student Related

Gender, type of school (public or private), class grade (8th, 9th, and 10th) and academic performance.

In Pakistan, public and private schools may differ in several aspects including fee structures, class strength and difficulty levels of internal examinations, with private schools being more expensive, with fewer students per classroom, and subjectively tougher internal examinations.

The academic performance was judged as the overall grade (a combination of all subjects including English, Mathematics and Science) in the latest internal examinations sat by the student as A+, A, B, C, or D.

#### Family Related

Family structure and type of accommodation (rented or owned).

#### Parent Related

Information on living status, education level, employment status, employment type and parenting styles was obtained from the student separately for the father and mother.

### Quantitative Variables

#### Academic Performance

The grades A+, A were categorized as “high” grades and grades B, C, and D were categorized as “low” grades.

#### Socio-Economic Status

We used variables which adolescent students are expected to have knowledge of to calculate a score which categorized students as belonging to either a high or low socioeconomic status. The points assigned to each variable are show in Table 1.

**TABLE 1:** Calculation of an estimated socioeconomic status.

**Variables**	**Points**	
School type	Public = 0	Private = 1
Family structure	Joint = 0	Nuclear = 1
Father’s employment status	No = 0	Yes = 1
Accommodation status	Rented = 0	Owned = 2
Father’s job type	Unemployed = 0	Government sector = 1 Private sector = 2
Mother’s employment status	Unemployed = 0	Employed = 1

*High SES, score ≥ 6; Low SES, score < 6; SES, socioeconomic status.*

#### Parenting Styles

**FIGURE 1:**
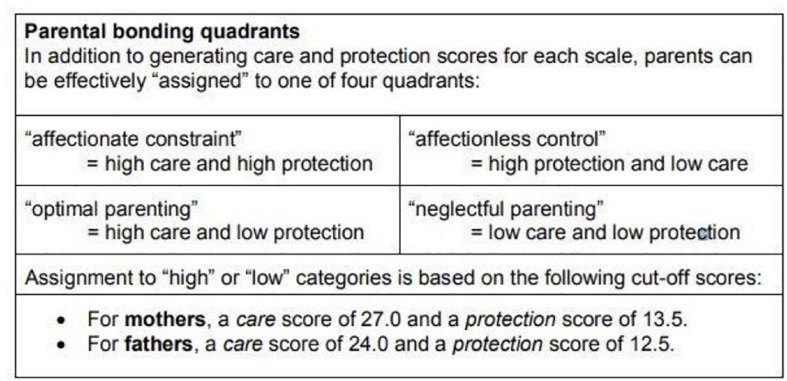
Assigining parenting styles using the PBI (Parker, 1979 #192).

The PBI is a 25 item questionnaire, with 12 items measuring “care” and 13 items measuring “overprotection.” All responses have a 4 point Likert scale ranging from 0 (very unlikely) to 3 (very likely). The responses are summed up to categorize each parent to exhibit low or high “care” and low or high “overprotection.” Based on these findings, each parent can then be put into one of the 4 quadrants representing parenting styles including “affectionate constraint,” “affectionless control,” “optimal parenting,” and “neglectful parenting.” This computation is explained in Figure 1 obtained from the information provided with the PBI (Parker et al., 1979).

### Bias

Students were allowed to fill in the *pro forma* and questionnaire independently to avoid bias during the data collection process. However, self-reporting of grades in latest examination may be subject to recall bias.

### Statistical Methods

Statistical analysis was performed using SPSS v.23 (IBM Corp., Armonk, NY, United States). Descriptive analyses were conducted on all study variables including socio-demographic factors and parenting styles. Categorical variables were reported as proportions and continuous variables as measures of central tendency. All continuous variables were subjected to a normality test. Mean and median values were reported for variables with normally distributed and skewed data, respectively.

The summary *t*-test was used to study the differences between mean “care” and “overprotection” scores of fathers and mothers. The independent sample *t*-test was used to study the factors associated with “care” and “overprotection” scores of fathers and mothers. Threshold for significance was *p* = 0.05.

The determinants of high grades including the influence of parenting styles were assessed using regression analysis. The outcome variable, student grades, was treated as binary (high grades and low grades). The threshold for statistical significance was *p* = 0.05. Crude Odds Ratios were adjusted for gender, school type, socioeconomic status, family structure, class grade, parents’ employments and education status.

### Ethics Statement

The study was approved by the Ethical Committee of the Khyber Medical University, Advance Studies and Research Board (KMU-AS&RB) in August 2016. Identifying information of students was not obtained. Permissions were obtained from the relevant authorities in the school administration before approaching the students and their parents. Written consent was obtained from the parents through the home-work diary of the students and verbal assent of each student was obtained.

## Results

### Participants and Descriptive Data

A total of 456 students were interviewed, with 249 (54.6%) males and 207 (45.4%) females. The majority (52.5%) were students of grade 8. Despite including an equal number of public and private schools, 63.6% of the students belonged to a public sector school. The reason may be due to the larger class strength in public schools in comparison to private schools. The nuclear family structure was dominant (64.3%), with most students living in rented accommodation (70.4%) with 42.8% reporting to have obtained high grades (A plus or A) in their latest internal examinations (Table 2).

**TABLE 2:** Participant and descriptive data.

**Participant characteristics (*n* = 456)**			***N* (%)**	**Response rate (%)**
Gender		Male	249(54.6%)	100%
		Female	207(45.4%)	
Class grade		Grade 8th	238(52.2%)	100%
		Grade 9th	110(24.1%)	
		Grade 10th	108(23.7%)	
Type of school		Public	290(63.6%)	100%
		Private	166(36.4%)	
Father	Living status	Alive	442(96.9%)	100%
		Deceased	14(3.1%)	
	Education level	Masters (Post-graduate)	8(1.8%)	45.6%
		Bachelors (Undergraduate)	7(1.5%)	
		FSC/FA (Grade 12)	27(5.9%)	
		Metric (Grade 10)	46(10.1%)	
		Middle school (Grade 7)	18(3.9%)	
		Below middle school or no education	102(22.4%)	
	Employment status	Employed	418(91.7%)	100%
		Unemployed^∗^	38(8.3%)	
	Type of employment	Government job	174(38.2%)	99%
		Private	176(38.6%)	
		Business	57(12.5%)	
		Don’t know	49(10.7%)	
Mother	Living status	Alive	456(100%)	100%
		Deceased	0(0%)	
	Education level	Masters (Post-graduate)	35(7.7%)	99.5%
		Bachelors (Undergraduate)	40(8.8%)	
		FSC/FA (Grade 12)	44(9.6%)	
		Metric (Grade 10)	56(12.3%)	
		Middle school (Grade 7)	37(8.1%)	
		Below middle school or no education	244(53.5%)	
	Employment status	Employed	51(11.2%)	100%
		Unemployed	405(88.8%)	
Family structure		Nuclear family	293(64.3%)	100%
		Joint family	163(35.7%)	
Accommodation status		Rented	321(70.4%)	100%
		Owned	135(29.6%)	
Academic performance		High grades (A+ and A Grades)	195(42.8%)	100%
		Low grades (Grades B, C, and D)	261(57.2%)	

*^∗^Includes fathers who are deceased.*

Majority of the students had both parents alive at the time of the interview. While all students’ mothers were alive, 14 students reported their father to have passed away. Surprisingly, only 46% of the students were able to report their father’s level of education compared to 99.5% for their mother. 9.2% of students reported their father to have an education level of grade 12 or above compared to 26% regarding their mother’s qualification. This was in contrast to 90% of the fathers being employed compared to only 11% of the mothers (Table 2).

**FIGURE 2:**
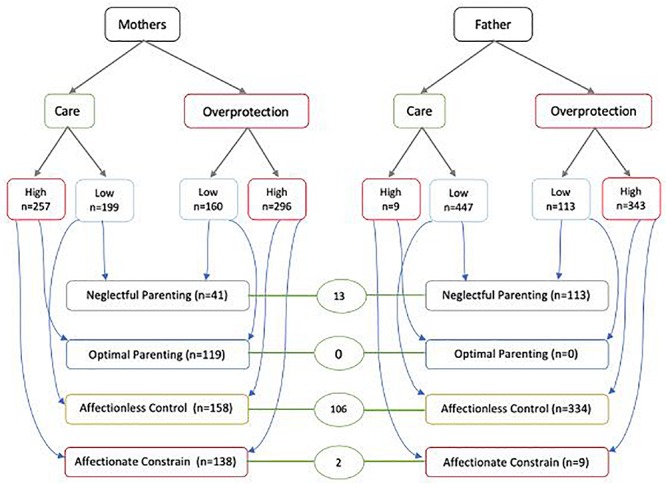
“Care,” “overprotection” and parenting styles for fathers and mothers as reported by students (*n* = 456). Green circles represent students with both parents showing the same parenting style – none of the students received “Optimal parenting” from both parents while 106 students received affectionless control from both parents.

A Total of 257 (56%) students reported their mother to exhibit a high level of “care” vs. only 9 (2%) students reporting the same for their father. In terms of “overprotection,” 343 (75%) and 296 (65%) students reported a high level for their father and mother, respectively. Based on combinations of these measures, the most common parenting style for both fathers (73%) and mothers (35%) was affectionless control and the least common for fathers was optimal parenting (0%) and neglectful parenting for mothers (9%). 121 (26%) students had both parents with the same parenting style, with 23% students having both parents show affectionless control and not a single student with both parents showing optimal parenting (Figure 2).

#### Determinants of High Grades

Our results show that high socioeconomic status [adjusted OR 2.78 (1.03, 7.52)], father’s education level till undergrad or above [adjusted OR 4.58 (1.49, 14.09)], father’s high “care” [adjusted OR 1.09 (1.01, 1.18)] and father’s affectionless control style of parenting [adjusted OR 3.23 (1.30, 8.03)] are significant factors contributing to high grades (Table 3).

**TABLE 3:** Academic performance: Determinants of “high” grades in the latest internal examinations.

**Variables**		**Crude Odds Ratio (95% CI)**	***p-value***	**Adjusted^∗^ Odds Ratio (95% CI)**	***p-value***
Gender	Male	1	0.215	1	0.156
	Female	0.79 (0.53, 1.15)		1.77 (0.80, 3.91)	
School type	Private	1	¡0.001	1	0.062
	Public	5.02 (3.22, 7.85)		2.15 (0.96, 4.80)	
Class grade	Grade 8	1	0.019	1	0.381
	Grade 9	1.61 (1.02, 2.55)		1.93 (0.75, 4.94)	
	Grade 10	1.80 (1.14, 2.85)		1.03 (0.37, 2.84)	
SES	Low	1	0.723	1	**0.045**
	High	1.10 (0.65, 1.87)		2.78 (1.03, 7.52)	
Family structure	Joint family	1	0.650	1	0.473
	Nuclear family	0.91 (0.62, 1.35)		0.78 (0.39, 1.55)	
Father’s education level	Completed middle school or below	1	¡0.001	1	**0.023**
	Completed metric or FSC/FA	2.77 (1.40, 5.45)		1.95 (0.90, 4.22)	
	Completed undergrad or Post-grad	10.00 (4.20, 23.83)		4.58 (1.49, 14.09)	
Mother’s education level	Completed middle school or below	1	¡0.001	1	0.066
	Completed metric or FSC/FA	3.03 (1.89, 4.86)		2.98 (0.97, 9.17)	
	Completed undergrad or Post-grad	2.19 (1.31, 3.66)		4.25 (0.94, 19.29)	
Father’s care	Low care	1	¡0.001	1	**0.024**
	High care	1.09 (1.04, 1.14)		1.09 (1.01, 1.18)	
Father’s overprotection	Low overprotection	1	0.028	1	0.420
	High overprotection	1.04 (1.01, 1.07)		1.02 (0.97, 1.08)	
Mother’s care	Low care	1	0.774	1	0.920
	High care	0.99 (0.96, 1.03)		1.01 (0.93, 1.08)	
Mother’s overprotection	Low overprotection	1	¡0.001	1	0.098
	High overprotection	1.07 (1.03, 1.11)		1.06 (0.99, 1.13)	
Father’s employment status	Unemployed	1	0.442	1	0.308
	Employed	1.31 (0.66, 2.60)		0.55 (0.17, 1.74)	
Mother’s employment status	Unemployed	1	0.370	1	0.790
	Employed	1.33 (0.74, 2.39)		0.85 (0.27, 2.72)	
Father’s parenting style	Neglectful parenting	1		1	
	Optimal parenting	–	–	–	–
	Affectionless control	1.74 (1.11, 2.72)	0.016	3.23 (1.30, 8.03)	**0.012**
	Affectionate constrain	2.57 (0.65, 10.13)	0.178	1.07 (0.16, 7.04)	0.941
Mother’s parenting style	Neglectful parenting	1	0.007	1	
	Optimal parenting	1.43 (0.65, 3,15)	0.370	1.04 (0.24, 4.54)	0.957
	Affectionless control	2.28 (1.07, 4.88)	0.033	0.99 (0.21, 4.62)	0.990
	Affectionate constrain	2.89 (1.34, 6.23)	0.007	2.93 (0.68, 12.62)	0.150

*SES, socioeconomic status. ^∗^Adjusted for gender, class grade, school type, socioeconomic status, family structure, father, and mother education and employment status. The significant values are indicated in bold.*

### Differences in “Care” and “Overprotection” Between Fathers and Mothers

#### Care

The mean “care” score for mothers were significantly higher than fathers overall. The difference remained significant for male and female students, public and private schools, joint and nuclear family structures and low and high socioeconomic statuses (Table 4).

**TABLE 4:** Differences between mean “care” and “overprotection” scores between fathers and mothers.

			**Father**	**Mother (*n* = 456)**	***p*-value**
**Care**	**Overall** (*n* = 456)	High	9 (2%)	257 (56.4%)	
		Low	447 (98%)	199 (43.6%)	
		**Mean score**	**15.06 ± 4.42**	**26.42 ± 4.84**	**<0.001**
	**Male students** (*n* = 249)	High	6 (2.4%)	116 (46.6%)	
		Low	243 (97.6%)	133 (53.4%)	
		**Mean score**	**14.84 ± 4.58**	**25.35 ± 4.88**	**<0.001**
	**Female students** (*n* = 207)	High	3 (1.4%)	141 (68.1%)	
		Low	204 (98.6%)	66 (31.9%)	
		**Mean score**	**15.32 ± 4.22**	**27.70 ± 4.48**	**<0.001**
	**Private school**(*n* = 166)	High	1 (0.6%)	110 (66.3%)	
		Low	165 (99.4%)	56 (33.7%)	
		**Mean score**	**14.06 ± 4.60**	**27.45 ± 4.48**	**<0.001**
	**Public school** (*n* = 290)	High	8 (2.8%)	147 (50.7%)	
		Low	282 (97.2%)	143 (49.3%)	
		**Mean score**	**15.63 ± 4.22**	**25.83 ± 4.95**	**<0.001**
	**Nuclear family** (*n* = 293)	High	6 (2%)	160 (54.6%)	
		Low	287 (98%)	133 (45.4%)	
		**Mean score**	**15.25 ± 4.30**	**26.45 ± 4.62**	**<0.001**
	**Joint family** (*n* = 163)	High	3 (1.8%)	97 59.5%)	
		Low	160 (98.2%)	66 (40.5%)	
		**Mean score**	**14.73 ± 4.63**	**26.37 ± 5.24**	**<0.001**
	**Low SES**(*n* = 388)	High	8 (2.1%)	220 (56.7%)	
		Low	380 (97.9%)	168 (43.3%)	
		**Mean score**	**14.99 ± 4.40**	**26.47 ± 4.90**	**<0.001**
	**High SES**(*n* = 65)	High	1 (1.5%)	36 (55.4%)	
		Low	64 (98.5%)	29 (44.6%)	
		**Mean score**	**15.57 ± 4.61**	**26.14 ± 4.53**	**<0.001**
**Overprotection**	**Overall**(*n* = 457)	High	343 (75.2%)	296 (64.9%)	
		Low	113 (24.8%)	160 (35.1%)	
		**Mean score**	**16.79**	**15.41**	**<0.001**
	**Male students**(*n* = 249)	High	181 (72.7%)	188 (75.5%)	
		Low	68 (27.3%)	61 (24.5%)	
		**Mean score**	**16.84 ± 6.39**	**16.87 ± 5.30**	0.957
	**Female students**(*n* = 207)	High	162 (78.3%)	108 (52.2%)	
		Low	45 (21.7%)	99 (47.8%)	
		**Mean score**	**16.72 ± 5.22**	**13.67 ± 5.59**	**<0.001**
	**Private school**(*n* = 166)	High	124 (74.7%)	75 (45.2%)	
		Low	42 (25.3%)	91 (54.8%)	
		**Mean score**	**16.62 ± 5.93**	**13.12 ± 5.42**	**<0.001**
	**Public school**(*n* = 290)	High	219 (75.5%)	221 (76.2%)	
		Low	71 (24.5%)	69 23.8%)	
		**Mean score**	**16.88 ± 5.86**	**16.73 ± 5.37**	0.748
	**Nuclear family (*n* = 293)**	High	228 (77.8%)	188 (64.2%)	
		Low	65 (22.2%)	105 (35.8%)	
		**Mean score**	**16.87 ± 5.71**	**15.45 ± 5.53**	**0.002**
	**Joint family (*n* = 163)**	High	115 (70.6%)	108 (66.3%)	
		Low	48 (29.4%)	55 (33.7%)	
		**Mean score**	**16.64 ± 6.19**	**15.35 ± 5.89**	0.055
	**Low SES (*n* = )**	High	289 (74.5%)	249 (64.2%)	
		Low	99 (25.5%)	139 (35.8%)	
		**Mean score**	**16.82 ± 5.85**	**15.36 ± 5.77**	**<0.001**
	**High SES (*n* = )**	High	51 (78.5%)	44 (67.7%)	
		Low	14 (21.5%)	21 (32.3%)	
		**Mean score**	**16.69 ± 6.22**	**15.66 ± 5.06**	0.302

*SES: socioeconomic status. The significant values are indicated in bold.*

#### Overprotection

The mean “overprotection” score was significantly higher for fathers overall. The difference remained significant for female students, private schools, nuclear family structure, and low socioeconomic status. However, there was no significant difference in mean “overprotection” scores between fathers and mothers for male students, public schools, joint family structures and high socioeconomic status (Table 4).

### Factors Associated With “Care” and “Overprotection” in Fathers and Mothers

#### Fathers

The mean “care” score was significantly higher for fathers as reported by children in public schools and with higher grades. There was no significant difference in mean care scores based on student gender, socioeconomic status or family structure (Table 5).

**TABLE 5:** Factors associated with “care” and “overprotection” for mothers and fathers.

	**Variables**	**Fathers**	***p*-value**	**Mothers**	***p*-value**
Care	Male students	14.84 ± 4.58	0.244	25.35 ± 4.88	**<0.001**
	Female students	15.32 ± 4.22		27.70 ± 4.48	
	Private school	14.06 ± 4.60	**0.001**	27.45 ± 4.48	**<0.001**
	Public school	15.63 ± 4.22		25.83 ± 4.95	
	Nuclear family	15.25 ± 4.30	0.230	26.45 ± 4.62	0.872
	Joint family	14.73 ± 4.63		26.37 ± 5.24	
	Low SES	14.99 ± 4.40	0.334	26.47 ± 4.90	0.609
	High SES	15.57 ± 4.61		26.14 ± 4.53	
	Low grades	14.36 ± 4.45	**<0.001**	26.48 ± 4.63	0.745
	High grades	16.00 ± 4.21		26.33 ± 5.12	
Overprotection	Male students	16.84 ± 6.39	0.833	16.87 ± 5.30	**<0.001**
	Female students	16.72 ± 5.22		13.67 ± 5.59	
	Private school	16.62 ± 5.93	0.647	13.12 ± 5.42	**<0.001**
	Public school	16.88 ± 5.86		16.73 ± 5.37	
	Nuclear family	16.87 ± 5.71	0.699	15.45 ± 5.53	0.855
	Joint family	16.64 ± 6.19		15.35 ± 5.89	
	Low SES	16.82 ± 5.85	0.872	15.36 ± 5.77	0.693
	High SES	16.69. ± 6.22		15.66 ± 5.06	
	Low grades	16.26 ± 5.95	**<0.027**	14.55 ± 5.61	**<0.001**
	High grades	17.49 ± 5.79		16.57 ± 5.52	

*SES, socioeconomic status. The significant values are indicated in bold.*

For “overprotection” the only factor associated with a significantly higher mean score was “high” grades (Table 5).

#### Mothers

A significantly higher mean “care” score for mothers was reported by female students and students in public schools. No significant differences were observed for the other factors (Table 5).

A significantly higher mean “overprotection” score was reported by male students, students in public schools and those with “high” grades for mothers (Table 5).

## Discussion

### Summary of Findings

Results of regression analysis show that socioeconomic status, father’s education level and fathers’ care scores have a significantly positive influence on the academic performance of adolescent students in Peshawar, Pakistan. The most common parenting style for both fathers and mothers was affectionless control. However, affectionless control exhibited by the father was the only parenting style significantly contributing to improved academic performance.

Overall, the mean “care” score was higher for mothers and the mean “overprotection” score was higher for fathers. However, differences in “overprotection” were eliminated for male students, public schooling, joint family structures and high socioeconomic status.

Public schooling was associated with a significantly higher mean “care” score for both fathers and mothers and a significantly higher mean “overprotection” score for mothers. High grades were associated with a significantly higher mean “overprotection” score for both fathers and mothers and a significantly higher mean “care” score for fathers. For mothers, female students reported a significantly higher mean care score and male students reported a significantly higher mean “overprotection” score.

An additional interesting finding from the results of the study was that only about half the students were able to report their father’s level of education compared to almost a 100% for their mother. From amongst those who did report, less than 10% of the father’s had an education level equal or above grade 12 compared to a quarter of the mothers. However, only 11% of the mothers were employed in contrast to 90% of the fathers.

### Previous Literature and Comparison of Main Findings

The results of our study have identified socioeconomic status, father’s education level and high care scores for fathers to be significant predictors of academic success in adolescent students. Previous literature has shown socioeconomic status to be a predictor of academic success (Gamoran, 1996; Sander, 1999; Lubienski and Lubienski, 2006).

Parental education has been frequently associated with improved academic performance (Dumka et al., 2008; Dubow et al., 2009; Masud et al., 2015). In 2011, a study by Farooq et al. described the factors affecting academic performance in 600 students at the secondary school level in a public school in Lahore, Pakistan. Results of their study also associate parental education level with academic success in students. However, their results are significant for the education level of the mother as well as the father. Additionally, they also reported significantly higher academic performance in females and in students belonging to a higher socioeconomic status, factors not significant in our study (Farooq et al., 2011). Differences may be explained by cultural variations in Lahore and Peshawar within Pakistan, which should be explored further.

The description of parenting styles and behaviors has evolved over the years. With some variation in terminologies, the essence lies in a few common principles. Diana Baumrind initially described three main parenting styles based on variations in normal parenting behaviors: authoritative, authoritarian and permissive (Baumrind, 1966, 1967). Building on the concepts put forth by Baumrind, Maccoby and Martin identified two dimensions, “responsiveness” and “demandingness,” which could classify parenting styles into 4 types, three of those described by Baumrind with the addition of neglectful parenting (Maccoby et al., 1983). The two dimensions, “responsiveness” and “demandingness,” often referred to as “warmth” and “control” in literature (Lamborn et al., 1991; Tagliabue et al., 2014), are similar to the two measures, “care” and “overprotection” assessed by the parental bonding instrument (Parker et al., 1979; Parker, 1989; Dudley and Wisbey, 2000). Based on this, the authoritative, authoritarian, permissive and neglectful parenting styles described by Baumrind and Maccoby are similar to the affectionate constraint, affectionless control, optimal, and neglectful styles as classified by the parental bonding instrument, respectively (Baumrind, 1991; Cavedo and Parker, 1994).

Results of our study show that affectionless control, similar to the authoritarian style of parenting, adapted by the father is significantly associated with improved academic performance. This differs from the popularity of the authoritative parenting style, similar to affectionate constraint, in determining academic success in literature from western cultures (Steinberg et al., 1989, 1992; Deslandes et al., 1998; Aunola et al., 2000; Adeyemo, 2005; Masud et al., 2015; Pinquart, 2016; Checa et al., 2019). Evidence from societies with cultural similarities with Pakistan presents varied findings. A study from Iran shows support for the authoritarian parenting style similar to our study (Rahimpour et al., 2015). A review of 39 studies published by Masud et al. (2015) in 2015 assesses the effect of parenting styles on academic performance (Masud et al., 2015 #205). The review very aptly described how the authoritative parenting style is the dominant and most effective style in terms of determining academic performance in the West and European countries while Asian cultures show more promising results for academic success for the authoritarian style (Dornbusch et al., 1987; Lin and Fu, 1990; Masud et al., 2015). The results of our study are in synchrony with these findings. However, our results also show that high father’s “care” scores are significant contributors to higher academic grades. Since no father showed optimal parenting and only 9 fathers had affectionate constraint, both parenting styles with high care scores, these results may be a reflection of the importance of father’s role in determining academic performance in Asian cultures. Findings supporting the authoritarian/affectionless control style may be due to the abundance of this parenting style. Perhaps a fairer comparison may be possible with a larger sample population with fathers showing all types of parenting styles equally.

### Interpretation and Explanation of Other Findings

Observations of factors associated with and differences in “care” and “overprotection” between fathers and mothers may be attributed to reverse causality and should be used as hypothesis generating.

Our results show that mothers have higher mean “care” score and fathers have a higher mean “overprotection” score. Since these scores are based on perceptions of the child, part of these observations may be explained by the cultural norms of expression of love and concern by fathers and mothers. With the difference in “overprotection” being eliminated for male and female children, it is possible that mothers are more overprotective of their sons. Male gender preference in Pakistan may be an explanation for this (Qadir et al., 2011).

Our results show lower employment rates for women despite higher education levels. The finding of higher education levels for females compared to males does not agree with national data, which reports findings from rural areas as well where education opportunities are limited for females (Hussain, 2005; Chaudhry and Rahman, 2009). Our results provide a zoomed in look at an urban population, which may have progressed enough to improve women’s education but cultural norms, gender discrimination and lack of opportunity still prevent women from stepping into the workface (Chaudhry, 2007; Begum and Sheikh, 2011).

### Implications and Future Direction

The findings of our study may have implications for future research and policy making.

Affectionless control is associated with improved academic performance but further research investigating the effects of this style on other aspects of child development, particularly emotional and psychological health, is needed. Factors affecting care and overprotection need to be studied in more detail so that parenting workshops and interventions are tailored to our population. Results also suggest that fathers should play a stronger role in parenting of adolescent students. School policies should make it mandatory for both parents to attend parent-teacher meetings and assigned home activities should include both parents.

### Limitations

Since the study is based on the urban population of Peshawar, results may not be generalizable to the adolescent students of the country which includes large rural populations. Academic performance was judged on latest internal examinations, the marking criteria for which may vary across schools. The use of external examinations would have standardized grades across schools but limited the sample to students of grade 9 and 10.

### Conclusion

Our study concludes that socioeconomic status, father’s level of education and high care scores for fathers are associated with improved academic outcomes in adolescent students in Peshawar, Pakistan. Affectionless control is the most common parenting style as perceived by the students and when adapted by the father, contributes to better grades. Further research investigating the effects of demonstrating affectionless control on the emotional and psychological health of students needs to be conducted. Parenting workshops and school policies should include recommendations to increase involvement of fathers in the parenting of adolescent children.

## Data Availability Statement

Data collected and stored as part of this study is available upon reasonable request.

## Ethics Statement

The studies involving human participants were reviewed and approved by the Khyber Medical University. Written informed consent to participate in this study was provided by the participants’ legal guardian/next of kin.

## Author Contributions

SM contributed in conceiving, designing, data acquisition, grant submission, and manuscript review. SHM involved in data analysis and manuscript writing. NQ involved in manuscript writing. MK was the principal investigator and supervisor for the project. FK and SK contributed in literature review and data management. All authors proofread and agreed on the final draft and accept responsibility for the work.

## Conflict of Interest

The authors declare that the research was conducted in the absence of any commercial or financial relationships that could be construed as a potential conflict of interest.

## References

[B1] Abidin R. R. (1992). The determinants of parenting behavior. *J. Clin. Child Psychol.* 21 407–412. 10.1207/s15374424jccp2104_12

[B2] Adeyemo D. A. (2005). Parental involvement, interest in schooling and school environment as predictors of academic self-efficacy among fresh secondary school students in Oyo State, Nigeria. *Electron. J. Res. Educ. Psychol.* 3 163–180.

[B3] Aunola K. Stattin H. Nurmi J.-E. (2000). Parenting styles and adolescents’ achievement strategies. *J. Adolesc.* 23 205–222. 10.1006/jado.2000.0308 10831143

[B4] Battle J. (2002). Longitudinal analysis of academic achievement amonga nationwide sample of hispanic students in one-versus dual-parent households. *Hisp. J. Behav. Sci.* 24 430–447. 10.1177/0739986302238213

[B5] Baumrind D. (1966). Effects of authoritative parental control on child behavior. *Child Dev.* 37 887–907. 10.1111/j.1467-8624.1966.tb05416.x

[B6] Baumrind D. (1967). Child care practices anteceding three patterns of preschool behavior. *Genet. Psychol. Monogr.* 75 43–88. 6032134

[B7] Baumrind D. (1991). The influence of parenting style on adolescent competence and substance use. *J. Early Adolesc.* 11 56–95. 10.1177/0272431691111004

[B8] Bean R. A. Barber B. K. Crane D. R. (2006). Parental support, behavioral control, and psychological control among African American youth: the relationships to academic grades, delinquency, and depression. *J. Fam. Issues* 27 1335–1355. 10.1177/0192513X06289649

[B9] Beauvais A. M. Stewart J. G. DeNisco S. Beauvais J. E. (2014). Factors related to academic success among nursing students: a descriptive correlational research study. *Nurse Educ. Today* 34 918–923. 10.1016/j.nedt.2013.12.005 24380623

[B10] Begum M. S. Sheikh Q. A. (2011). Employment situation of women in Pakistan. *Int. J. Soc. Econ.* 38 98–113. 10.1108/03068291111091981

[B11] Behzadi B. Parker G. (2015). A Persian version of the parental bonding instrument: factor structure and psychometric properties. *Psychiatry Res.* 225 580–587. 10.1016/j.psychres.2014.11.042 25530418

[B12] Belsky J. (1984). The determinants of parenting: a process model. *Child Dev.* 55 83–96. 10.2307/1129836 6705636

[B13] Bluestone C. Tamis-LeMonda C. S. (1999). Correlates of parenting styles in predominantly working- and middle-class African American mothers. *J. Marriage Fam.* 61 881–893. 10.2307/354010

[B14] Cavedo L. Parker G. (1994). Parental bonding instrument. *Soc. Psychiatr. Psychiatr. Epidemiol.* 29 78–82. 8009323

[B15] Chaudhry I. S. (2007). Gender inequality in education and economic growth: case study of Pakistan. *Pakistan Horizon* 60 81–91. 12289815

[B16] Chaudhry I. S. Rahman S. (2009). The impact of gender inequality in education on rural poverty in Pakistan: an empirical analysis. *Eur. J. Econ. Finance Adm. Sci.* 15 174–188.

[B17] Checa P. Abundis-Gutierrez A. (2017). Parenting and temperament influence on school success in 9–13 year olds. *Front. Psychol.* 8:543. 10.3389/fpsyg.2017.00543 28446886 PMC5388739

[B18] Checa P. Abundis-Gutierrez A. Pérez-Dueñas C. Fernández-Parra A. (2019). Influence of maternal and paternal parenting style and behavior problems on academic outcomes in primary school. *Front. Psychol.* 10:378. 10.3389/fpsyg.2019.00378 30881327 PMC6405423

[B19] Chow H. P. (2000). The determinants of academic performance: Hong Kong immigrant students in Canadian schools. *Can. Ethn. Stud. J.* 32 105–105.

[B100] Cohen D. A. Rice J. (1997). Parenting styles, adolescent substance use, and academic achievement. *J. Drug Educ.* 27 199–211. 10.2190/QPQQ-6Q1GUF7D-5UTJ 9270213

[B20] Cutrona C. E. Cole V. Colangelo N. Assouline S. G. Russell D. W. (1994). Perceived parental social support and academic achievement: an attachment theory perspective. *J. Pers. Soc. Psychol.* 66 369–378. 10.1037/0022-3514.66.2.369 8195992

[B21] Dennis J. M. Phinney J. S. Chuateco L. I. (2005). The role of motivation, parental support, and peer support in the academic success of ethnic minority first-generation college students. *J. Coll. Stud. Dev.* 46 223–236. 10.1353/csd.2005.0023

[B22] Desimone L. (1999). Linking parent involvement with student achievement: do race and income matter? *J. Educ. Res.* 93 11–30. 10.1080/00220679909597625

[B23] Deslandes R. Bouchard P. St-Amant J.-C. (1998). Family variables as predictors of school achievement: sex differences in Quebec adolescents. *Can. J. Educ.* 23 390–404.

[B24] Deslandes R. Royer E. Turcotte D. Bertrand R. (1997). School achievement at the secondary level: influence of parenting style and parent involvement in schooling. *McGill J. Educ.* 32

[B25] Dornbusch S. M. Ritter P. L. Leiderman P. H. Roberts D. F. Fraleigh M. J. (1987). The relation of parenting style to adolescent school performance. *Child Dev.* 58 1244–1257. 10.1111/j.1467-8624.1987.tb01455.x 3665643

[B26] Dubow E. F. Boxer P. Huesmann L. R. (2009). Long-term effects of parents’ education on children’s educational and occupational success: mediation by family interactions, child aggression, and teenage aspirations. *Merrill Palmer Q.* 55 224–249. 10.1353/mpq.0.0030 20390050 PMC2853053

[B27] Dudley R. L. Wisbey R. L. (2000). The relationship of parenting styles to commitment to the church among young adults. *Relig. Educ.* 95 38–50. 10.1080/0034408000950105

[B28] Dumka L. E. Gonzales N. A. Bonds D. D. Millsap R. E. (2008). Academic success of Mexican origin adolescent boys and girls: the role of mothers’ and fathers’ parenting and cultural orientation. *Sex Roles* 60 588–599. 10.1007/s11199-008-9518-z 21731172 PMC3128498

[B29] Farooq M. S. Chaudhry A. H. Shafiq M. Berhanu G. (2011). Factors affecting students’ quality of academic performance: a case of secondary school level. *J. Qual. Technol. Manag.* 7 1–14.

[B30] Farsides T. Woodfield R. (2003). Individual differences and undergraduate academic success: the roles of personality, intelligence, and application. *Pers. Individ. Differ.* 34 1225–1243. 10.1016/S0191-8869(02)00111-3

[B31] Flynn D. M. MacLeod S. (2015). Determinants of Happiness in Undergraduate University Students. *Coll. Stud. J.* 49 452–460.

[B32] Gamoran A. (1996). Student achievement in public magnet, public comprehensive, and private city high schools. *Educ. Eval. Policy Anal.* 18 1–18. 10.3102/01623737018001001

[B33] Ginsburg G. S. Bronstein P. (1993). Family factors related to children’s intrinsic/extrinsic motivational orientation and academic performance. *Child Dev.* 64 1461–1474. 10.1111/j.1467-8624.1993.tb02964.x 8222884

[B34] Hawkins J. D. (1997). “Academic performance and school success: sources and consequences,” in *Healthy Children 2010: Enhancing Children’s Wellness*, eds Weissberg R. P. Gullotta T. P. Hampton R. L. Ryan B. A. Adams G. R., (Thousand Oaks, CA: Sage Publications, Inc), 278–305.

[B35] Hillstrom K. A. (2009). *Are Acculturation and Parenting Styles Related to Academic Achievement Among Latino students?* dissertation, University of Southern California, Los Angeles, CA.

[B36] Hussain I. (2005). “Education, employment and economic development in Pakistan,” in *Education Reform in Pakistan: Building for the Future*, ed. Hathaway R. M., (Washington, DC: Woodrow Wilson International Center for Scholars), 33–45.

[B37] Huver R. M. E. Otten R. de Vries H. Engels R. C. (2010). Personality and parenting style in parents of adolescents. *J. Adolesc.* 33 395–402. 10.1016/j.adolescence.2009.07.012 19716597

[B38] Jeynes W. H. (2007). The relationship between parental involvement and urban secondary school student academic achievement: a meta-analysis. *Urban Educ.* 42 82–110. 10.1177/0042085906293818

[B39] Khalid A. Qadir F. Chan S. W. Schwannauer M. (2018). Parental bonding and adolescents’ depressive and anxious symptoms in Pakistan. *J. Affect. Disord.* 228 60–67. 10.1016/j.jad.2017.11.050 29232565

[B40] Kuh G. D. Kinzie J. L. Buckley J. A. Bridges B. K. Hayek J. C. (2006). *What Matters to Student Success: A Review of the Literature.* Washington, DC: National Postsecondary Education Cooperative.

[B41] Lamborn S. D. Mounts N. S. Steinberg L. Dornbusch S. M. (1991). Patterns of competence and adjustment among adolescents from authoritative, authoritarian, indulgent, and neglectful families. *Child Dev.* 62 1049–1065. 10.1111/j.1467-8624.1991.tb01588.x 1756655

[B42] Lin C. Y. C. Fu V. R. (1990). A comparison of child-rearing practices among Chinese, immigrant Chinese, and Caucasian-American parents. *Child Dev.* 61 429–433. 10.1111/j.1467-8624.1990.tb02789.x

[B43] Linnenbrink E. A. Pintrich P. R. (2002). Motivation as an enabler for academic success. *Sch. Psychol. Rev.* 31 313–327. 10.1080/17483107.2018.1471169 29772940

[B44] Liu J. Li L. Fang F. (2011). Psychometric properties of the Chinese version of the Parental Bonding Instrument. *Int. J. Nurs. Stud.* 48 582–589. 10.1016/j.ijnurstu.2010.10.008 21094942 PMC3080463

[B45] Lubienski C. Lubienski S. (2006). *Charter, Private, Public Schools and Academic Achievement: New Evidence from NAEP Mathematics Data.* New York, NY: Columbia University.

[B46] Maccoby E. Martin J. Hetherington E. Mussen P. (1983). “Socialization in the context of the family: Parent-child interaction,” in *Handbook of Child Psychology: Socialization, Personality, and Social Development*, 4th Edn, Vol. 4 ed. Hetherington E. M., (Hoboken. NJ: John Wiley & Sons), 101.

[B47] Masud H. Thurasamy R. Ahmad M. S. (2015). Parenting styles and academic achievement of young adolescents: a systematic literature review. *Qual. Quant.* 49 2411–2433. 10.1007/s11135-014-0120-x

[B48] McCabe J. E. (2014). Maternal personality and psychopathology as determinants of parenting behavior: a quantitative integration of two parenting literatures. *Psychol. Bull.* 140 722–750. 10.1037/a0034835 24295555

[B49] McClelland M. M. Morrison F. J. Holmes D. L. (2000). Children at risk for early academic problems: the role of learning-related social skills. *Early Child. Res. Q.* 15 307–329. 10.1016/S0885-2006(00)00069-7

[B50] Mujtaba T. Furnham A. (2001). A cross-cultural study of parental conflict and eating disorders in a non-clinical sample. *Int. J. Soc. Psychiatry* 47 24–35. 10.1177/002076400104700103 11322404

[B51] Murphy E. Wickramaratne P. Weissman M. (2010). The stability of parental bonding reports: a 20-year follow-up. *J. Affect. Disord.* 125 307–315. 10.1016/j.jad.2010.01.003 20138671 PMC2889015

[B52] Osorio A. González-Cámara M. (2016). Testing the alleged superiority of the indulgent parenting style among Spanish adolescents. *Psicothema* 28 414–420. 27776610 10.7334/psicothema2015.314

[B53] Östberg M. Hagekull B. (2000). A structural modeling approach to the understanding of parenting stress. *J. Clin. Child Psychol.* 29 615–625. 10.1207/S15374424JCCP2904_13 11126638

[B54] Parker G. (1979). Reported parental characteristics in relation to trait depression and anxiety levels in a non-clinical group. *Aust. N. Z. J. Psychiatry* 13 260–264. 10.3109/00048677909159146 293182

[B55] Parker G. (1983). *Parental Overprotection: A Risk Factor in Psychosocial Development.* New York, NY: Grune & Stratton.

[B56] Parker G. (1989). The parental bonding instrument: psychometric properties reviewed. *Psychiatr. Dev.* 7 317–335. 2487899

[B57] Parker G. (1990). The parental bonding instrument. *Soc. Psychiatr. Psychiatr. Epidemiol.* 25 281–282. 10.1007/BF00782881 2291129

[B58] Parker G. Tupling H. Brown L. B. (1979). A parental bonding instrument. *Br. J. Med. Psychol.* 52 1–10.

[B59] Parsasirat Z. Montazeri M. Yusooff F. Subhi N. Nen S. (2013). The most effective kinds of parents on children’s academic achievement. *Asian Soc. Sci.* 9 229–242.

[B60] Pinquart M. (2016). Associations of parenting styles and dimensions with academic achievement in children and adolescents: a meta-analysis. *Educ. Psychol. Rev.* 28 475–493. 10.1007/s10648-015-9338-y

[B61] Ponnet K. Mortelmans D. Wouters E. Van Leeuwen K. Bastaits K. Pasteels I. (2013). Parenting stress and marital relationship as determinants of mothers’ and fathers’ parenting. *Pers. Relat.* 20 259–276. 10.1111/j.1475-6811.2012.01404.x

[B62] Qadir F. Khan M. M. Medhin G. Prince M. (2011). Male gender preference, female gender disadvantage as risk factors for psychological morbidity in Pakistani women of childbearing age - a life course perspective. *BMC Public Health* 11:745. 10.1186/1471-2458-11-745 21958069 PMC3195096

[B63] Qadir F. Stewart R. Khan M. Prince M. (2005). The validity of the Parental Bonding Instrument as a measure of maternal bonding among young Pakistani women. *Soc. Psychiatr. Psychiatr. Epidemiol.* 40 276–282. 10.1007/s00127-005-0887-0 15834778

[B64] Rahimpour P. Direkvand-Moghadam A. Direkvand-Moghadam A. Hashemian A. (2015). Relationship between the parenting styles and students’ educational performance among Iranian girl high school students, a cross- sectional study. *J. Clin. Diagn. Res.* 9:JC05–JC07. 10.7860/JCDR/2015/15981.6914 26813692 PMC4717796

[B65] Sander W. (1999). Private schools and public school achievement. *J. Hum. Resour.* 34 697–709. 10.2307/146413

[B66] Sanders M. G. (1998). The effects of school, family, and community support on the academic achievement of African American adolescents. *Urban Educ.* 33 385–409. 10.1177/0042085998033003005

[B67] Shute V. J. Hansen E. G. Underwood J. S. Razzouk R. (2011). A review of the relationship between parental involvement and secondary school students’ academic achievement. *Educ. Res. Int.* 2011:915326.

[B68] Simons R. L. Beaman J. Conger R. D. Chao W. (1993). Childhood experience, conceptions of parenting, and attitudes of spouse as determinants of parental behavior. *J. Marriage Fam.* 55 91–106. 10.2307/352961

[B69] Simons R. L. Whitbeck L. B. Conger R. D. Melby J. N. (1990). Husband and wife differences in determinants of parenting: a social learning and exchange model of parental behavior. *J. Marriage Fam.* 52 375–392. 10.2307/353033

[B70] Smith C. L. (2010). Multiple determinants of parenting: predicting individual differences in maternal parenting behavior with toddlers. *Parenting* 10 1–17. 10.1080/15295190903014588

[B71] Steinberg L. Elmen J. D. Mounts N. S. (1989). Authoritative parenting, psychosocial maturity, and academic success among adolescents. *Child Dev.* 60 1424–1436. 10.2307/1130932 2612251

[B72] Steinberg L. Lamborn S. D. Dornbusch S. M. Darling N. (1992). Impact of parenting practices on adolescent achievement: authoritative parenting, school involvement, and encouragement to succeed. *Child Dev.* 63 1266–1281. 10.1111/j.1467-8624.1992.tb01694.x 1446552

[B73] Tagliabue S. Olivari M. G. Bacchini D. Affuso G. Confalonieri E. (2014). Measuring adolescents’ perceptions of parenting style during childhood: psychometric properties of the parenting styles and dimensions questionnaire. *Psicol. Teoria e Pesquisa* 30 251–258. 10.1590/s0102-37722014000300002

[B74] Tomul E. Savasci H. S. (2012). Socioeconomic determinants of academic achievement. *Educ. Assess. Eval. Account.* 24 175–187. 10.1007/s11092-012-9149-9143

[B75] Vacha E. F. McLaughlin T. F. (1992). The social structural, family, school, and personal characteristics of at-risk students: policy recommendations for school personnel. *J. Educ.* 174 9–25. 10.1177/002205749217400303

[B76] Valiente C. Lemery-Chalfant K. Castro K. S. (2007). Children’s effortful control and academic competence: mediation through school liking. *Merrill Palmer Q.* 53 1–25. 10.1353/mpq.2007.0006

[B77] Vandenbroucke J. P. von Elm E. Altman D. G. Gøtzsche P. C. Mulrow C. D. Pocock S. J. (2014). Strengthening the Reporting of Observational Studies in Epidemiology (STROBE): explanation and elaboration. *Int. J. Surg.* 12 1500–1524. 25046751 10.1016/j.ijsu.2014.07.014

[B78] Wang Q. Leichtman M. D. (2000). Same beginnings, different stories: a comparison of American and Chinese children’s narratives. *Child Dev.* 71 1329–1346. 10.1111/1467-8624.00231 11108099

[B79] Weis M. Trommsdorff G. Muñoz L. (2016). Children’s self-regulation and school achievement in cultural contexts: the role of maternal restrictive control. *Front. Psychol.* 7:722. 10.3389/fpsyg.2016.00722 27303318 PMC4885849

[B80] Weiss L. H. Schwarz J. C. (1996). The relationship between parenting types and older adolescents’ personality, academic achievement, adjustment, and substance use. *Child Dev.* 67 2101–2114. 10.1111/j.1467-8624.1996.tb01846.x 9022232

[B81] Wilhelm K. Niven H. Parker G. Hadzi-Pavlovic D. (2005). The stability of the parental bonding instrument over a 20-year period. *Psychol. Med.* 35 387–393. 10.1017/s0033291704003538 15841874

[B82] York T. Gibson C. Rankin S. (2015). Defining and measuring academic success. *Pract. Assess. Res. Eval.* 20 1–20.

[B83] Zahedani Z. Z. Rezaee R. Yazdani Z. Bagheri S. Nabeiei P. (2016). The influence of parenting style on academic achievement and career path. *J. Adv. Med. Educ. Prof.* 4 130–134. 27382580 PMC4927255

[B84] Zakeri H. Karimpour M. (2011). Parenting styles and self-esteem. *Proc. Soc. Behav. Sci.* 29 758–761. 10.1016/j.sbspro.2011.11.302

[B85] Zhang X. Hu B. Y. Ren L. Huo S. Wang M. (2019). Young Chinese children’s academic skill development: identifying child-, family-, and school-level factors. *New Dir. Child Adolesc. Dev.* 2019 9–37. 10.1002/cad.20271 30615267

