# Supplementary material for: Academic Performance in Adolescent Students: The Role of Parenting Styles and Socio-Demographic Factors – A Cross Sectional Study From Peshawar, Pakistan
Source: Front Psychol. 2019 Nov 8;10:2497. doi: 10.3389/fpsyg.2019.02497 (PMC6856224; doi:10.3389/fpsyg.2019.02497)
Supplement: Supplementary file 1 [file Data_Sheet_1.PDF]

QUESTIONNAIRE:

Study

Instructions for participants

1. Kindly ask your instructor if you do not understand the question
2. Choose only one answer for one question
3. Do not leave any questions blank

GLOBAL SCHOOL BASED STUDENT HEALTH SURVEY (GSHS) 2016

Pakistan Questionnaire

Questions about demographics have been selected

1. What class are you in?	________________class
2. What is the type of your school?	a. Government<br>b. Private
3. What is your gender?	a. Male<br>b. Female
4. Choose the family setup you live in?	c. Independent family<br>d. Joint family with grandpaernts<br>e. Don't know<br>f. Other______
5. What is the status of your father?	a. Alive<br>b. Passed away

6. What is your father's education level?	a. Masters (MA/MSC)<br>b. Bachelors(BA/BSC)<br>c. College FSC<br>d. High school Matric<br>e. Middle school 8th<br>e. less or No education
6. What is your mother's education level?	a. Masters (MA/MSC)<br>b. Bachelors(BA/BSC)<br>c. College FSC<br>d. High school Matric<br>e. Middle school 8th<br>e. less or No education
8. What is your father's job status	a. Employed<br>b. Unemployed
7. What kind of job does your father have?	c. Government______<br>d. Private____________<br>e. Non-gvernment___<br>f. Don't know____
The house you are currently living in is?	a. Rental<br>b. owned
9. What is your mother's job status?	a. Employed<br>b. Unemployed
10. During the past 12 months how often have you felt lonely and sad?	a. Never<br>b. Rarely<br>c. Sometimes<br>d. Most of the time<br>e. Always
11. During the past 12 months how often have you been so worried about something that you could not sleep at night?	a. Never<br>b. Rarely<br>c. Sometimes<br>d. Most of the time<br>e. Always

Questions related to school grade.

12. What were your grades in your last promotion/ final term exam	a. A+ grade/ outstanding<br>b. A grade / Excellent<br>c. B Grade/ Average/ good<br>d. C grade/ Fair<br>e. D Grade/ Poor/ fail
