# Supplementary material for: Academic Performance in Adolescent Students: The Role of Parenting Styles and Socio-Demographic Factors – A Cross Sectional Study From Peshawar, Pakistan
Source: Front Psychol. 2019 Nov 8;10:2497. doi: 10.3389/fpsyg.2019.02497 (PMC6856224; doi:10.3389/fpsyg.2019.02497)
Supplement: Supplementary file 2 [file Data_Sheet_2.PDF]

PARENTAL BONDING INSTRUMENT (PBI)

BLACK DOG INSTITUTE

Authors
Gordon Parker, Hilary Tupling and L.B. Brown

Variables measured
Two scales termed 'care' and 'overprotection' or 'control', measure fundamental parental styles as perceived by the child. The measure is 'retrospective', meaning that adults (over 16 years) complete the measure for how they remember their parents during their first 16 years. The measure is to be completed for both mothers and fathers separately. There are 25 item questions, including 12 'care' items and 13 'overprotection' items.

Scoring instructions
Unlike the Intimate Bond Measure (IBM), not all items are scored in the same direction.

Care	
Items: 1, 5, 6, 11, 12, 17:	Very like = 3<br>Moderately like = 2<br>Moderately unlike = 1<br>Very unlike = 0
Items: 2, 4, 14, 16, 18, 24	Very unlike = 3<br>Moderately unlike = 2<br>Moderately like = 1<br>Very like = 0
Overprotection	
Items: 8, 9, 10, 13, 19, 20, 23	Very like = 3<br>Moderately like = 2<br>Moderately unlike = 1<br>Very unlike = 0
Items: 3, 7, 15, 21, 22, 25	Very unlike = 3<br>Moderately unlike = 2<br>Moderately like = 1<br>Very like = 0

This document may be freely downloaded and distributed on condition no change is made to the content. The information in this document is not intended as a substitute for professional medical advice, diagnosis or treatment. Not to be used for commercial purposes and not to be hosted electronically outside of the Black Dog Institute website. www.blackdoginstitute.org.au

**Parental bonding quadrants**<br>In addition to generating care and protection scores for each scale, parents can be effectively "assigned" to one of four quadrants:	
"affectionate constraint"<br>= high care and high protection	"affectionless control"<br>= high protection and low care
"optimal parenting"<br>= high care and low protection	"neglectful parenting"<br>= low care and low protection
Assignment to "high" or "low" categories is based on the following cut-off scores:	
• For **mothers**, a *care* score of 27.0 and a *protection* score of 13.5.<br>• For **fathers**, a *care* score of 24.0 and a *protection* score of 12.5.	

Populations measured

Original data [1] were generated from 150 subjects including students and nurses and 500 general practice attenders. Numerous other populations have been studied subsequently.

Reliability and validity

The PBI has been found to have good reliability and validity based on several studies.

In the original study [1] the PBI possessed good internal consistency and re-test reliability. Further reassuring data have been derived by examining the test-retest reliability of the PBI over extended periods, and we will shortly be publishing data for a 20-year interval. The PBI has been shown to have satisfactory construct and convergent validity and to be independent of mood effects [see 2].

Availability

A copy of the full 25-item forms for scoring mothers and fathers is attached below. Please follow the scoring instructions. The standard application asks subjects to score their biological parents (one for each form) as the subject remembers them in their first sixteen years. In some studies, other "parent figures" have and can clearly be rated.

A modified version of the PBI (the MOPS or Measure of Parenting Style) was developed in 1997 for two purposes. It overcame one of the PBI limitations in having some 'double negative' items, and which can cause some confusion. Thus, all items are constructed in a direct way. Secondly, while preserving the 'care' and 'control' scales, they are considerably reduced in terms of the numbers of items. Thirdly, there is an 'abuse' scale. Thus, the MOPS is described after the PBI measure.

The PBI is not held under copyright. Therefore, clinicians and researchers are free to use the measure without obtaining permission.

References

[1] Parker, G., Tupling, H., and Brown, L.B. (1979) A Parental Bonding Instrument. British Journal of Medical Psychology, 1979, 52, 1-10.

[2] Parker, G. (1983) Parental Overprotection: A Risk Factor in Psychosocial Development, Grune & Stratton, New York. [A monograph describing the development of the PBI and its application across a wide range of psychiatric conditions and other disorders, as well as validity studies]

This document may be freely downloaded and distributed on condition no change is made to the content. The information in this document is not intended as a substitute for professional medical advice, diagnosis or treatment. Not to be used for commercial purposes and not to be hosted electronically outside of the Black Dog Institute website. www.blackdoginstitute.org.au

MOTHER FORM

This questionnaire lists various attitudes and behaviours of parents. As you remember your MOTHER in your first 16 years would you place a tick in the most appropriate box next to each question.

	Very like	Moderately like	Moderately unlike	Very unlike
1. Spoke to me in a warm and friendly voice	☐	☐	☐	☐
2. Did not help me as much as I needed	☐	☐	☐	☐
3. Let me do those things I liked doing	☐	☐	☐	☐
4. Seemed emotionally cold to me	☐	☐	☐	☐
5. Appeared to understand my problems and worries	☐	☐	☐	☐
6. Was affectionate to me	☐	☐	☐	☐
7. Liked me to make my own decisions	☐	☐	☐	☐
8. Did not want me to grow up	☐	☐	☐	☐
9. Tried to control everything I did	☐	☐	☐	☐
10. Invaded my privacy	☐	☐	☐	☐
11. Enjoyed talking things over with me	☐	☐	☐	☐
12. Frequently smiled at me	☐	☐	☐	☐
13. Tended to baby me	☐	☐	☐	☐
14. Did not seem to understand what I needed or wanted	☐	☐	☐	☐
15. Let me decide things for myself	☐	☐	☐	☐
16. Made me feel I wasn't wanted	☐	☐	☐	☐
17. Could make me feel better when I was upset	☐	☐	☐	☐
18. Did not talk with me very much	☐	☐	☐	☐
19. Tried to make me feel dependent on her/him	☐	☐	☐	☐
20. Felt I could not look after myself unless she/he was around	☐	☐	☐	☐
21. Gave me as much freedom as I wanted	☐	☐	☐	☐
22. Let me go out as often as I wanted	☐	☐	☐	☐
23. Was overprotective of me	☐	☐	☐	☐
24. Did not praise me	☐	☐	☐	☐
25. Let me dress in any way I pleased	☐	☐	☐	☐

This document may be freely downloaded and distributed on condition no change is made to the content. The information in this document is not intended as a substitute for professional medical advice, diagnosis or treatment. Not to be used for commercial purposes and not to be hosted electronically outside of the Black Dog Institute website. www.blackdoginstitute.org.au

FATHER FORM

This questionnaire lists various attitudes and behaviours of parents. As you remember your FATHER in your first 16 years would you place a tick in the most appropriate box next to each question.

	Very like	Moderately like	Moderately unlike	Very unlike
1. Spoke to me in a warm and friendly voice	☐	☐	☐	☐
2. Did not help me as much as I needed	☐	☐	☐	☐
3. Let me do those things I liked doing	☐	☐	☐	☐
4. Seemed emotionally cold to me	☐	☐	☐	☐
5. Appeared to understand my problems and worries	☐	☐	☐	☐
6. Was affectionate to me	☐	☐	☐	☐
7. Liked me to make my own decisions	☐	☐	☐	☐
8. Did not want me to grow up	☐	☐	☐	☐
9. Tried to control everything I did	☐	☐	☐	☐
10. Invaded my privacy	☐	☐	☐	☐
11. Enjoyed talking things over with me	☐	☐	☐	☐
12. Frequently smiled at me	☐	☐	☐	☐
13. Tended to baby me	☐	☐	☐	☐
14. Did not seem to understand what I needed or wanted	☐	☐	☐	☐
15. Let me decide things for myself	☐	☐	☐	☐
16. Made me feel I wasn't wanted	☐	☐	☐	☐
17. Could make me feel better when I was upset	☐	☐	☐	☐
18. Did not talk with me very much	☐	☐	☐	☐
19. Tried to make me feel dependent of her/him	☐	☐	☐	☐
20. Felt I could not look after myself unless she/he was around	☐	☐	☐	☐
21. Gave me as much freedom as I wanted	☐	☐	☐	☐
22. Let me go out as often as I wanted	☐	☐	☐	☐
23. Was overprotective of me	☐	☐	☐	☐
24.Did not praise me	☐	☐	☐	☐
25. Let me dress in any way I pleased	☐	☐	☐	☐

This document may be freely downloaded and distributed on condition no change is made to the content. The information in this document is not intended as a substitute for professional medical advice, diagnosis or treatment. Not to be used for commercial purposes and not to be hosted electronically outside of the Black Dog Institute website. www.blackdoginstitute.org.au
