# Supplementary material for: Academic Performance in Adolescent Students: The Role of Parenting Styles and Socio-Demographic Factors – A Cross Sectional Study From Peshawar, Pakistan
Source: Front Psychol. 2019 Nov 8;10:2497. doi: 10.3389/fpsyg.2019.02497 (PMC6856224; doi:10.3389/fpsyg.2019.02497)
Supplement: Supplementary file 3 [file Data_Sheet_3.PDF]

QUESTIONNAIRE:

study.

Instructions for participants

1. Kindly ask your instructor if you do not understand the questions
2. Choose only one answer for one question.
3. Do not leave any questions blank.

GLOBAL SCHOOL BASED STUDENT HEALTH SURVEY (GSHS) 2016
Pakistan Questionnaire
Questions about demographics have been selected

1. What class are you in?<br>آپ کونسی کلاس میں پڑھتے ہیں ؟	_____________class	
2. What is the type of your school?<br>آپ کس قسم کے سکول میں پڑھتے ہیں ؟	a. Government<br>b. Private	سرکاری<br>غیر سرکاری
3. What is your gender?<br>آپکا جنس کیا ہے ؟	a. Male<br>b. Female	مرد<br>عورت
4. Choose the family setup you live in?<br>آپ کس قسم کی فیملی میں رہتے ہیں ؟	a. Independent family<br>b. Joint with grandparents<br>c. Don't know<br>d. Other__________	اکیلی فیملی<br>جائنٹ فیملی<br>نہیں معلوم<br>کوئی اور :
5. What is the status of your father?<br>آپکے باپ کے بارے میں سوال ؟	a. Alive<br>b. Passed away	زندہ<br>وفات

6. What is your father's education level?<br>آپکے والد کی تعلیم کیا ہے؟	a. Masters (MA/MSC) ماسٹرز<br>b. Bachelors(BA/BSC) بیچلرز<br>c. College FSC کالج<br>d. High school Matric میٹرک<br>e. Middle school 8th مڈل سکول<br>e. less or No education غیر تعلیم یافتہ
7. What is your mother's education level?<br>آپکی والدہ کی تعلیم کیا ہے؟	a. Masters (MA/MSC) ماسٹرز<br>b. Bachelors(BA/BSC) بیچلرز<br>c. College FSC کالج<br>d. High school Matric میٹرک<br>e. Middle school 8th مڈل سکول<br>e. less or No education غیر تعلیم یافتہ
8. What is your father's job status ?<br>آپکے والد کا روزگار کیا ہے؟	a. Employed روزگار یافتہ<br>b. Unemployed بے روزگار
9. What kind of job does your father have?<br>آپکے والد کس قسم کی نوکری کرتے ہیں	c. Government_____ سرکاری<br>d. Private_________ پرائیویٹ<br>e. Non-government__ غیر سرکاری<br>f. Don't know___ نہیں معلوم
10. The house you are currently living in is?<br>آپ جس گھر میں رہتے ہیں وہ ___ ہے؟	a. rental کرائے کا<br>b. Owned اپنا
11. What is your mother's job status?<br>آپکی والدہ کا روزگار کیا ہے؟	a. Employed روزگار یافتہ<br>b. Unemployed بے روزگار
12. During the past 12 months how often have you felt lonely and sad?<br>گزشتہ 12 مہینے میں آپکو کتنی مرتبہ اکیلا پن اور دکھ محسوس ہوا؟	a. Never کبھی نہیں<br>b. Rarely بہت کم<br>c. Sometimes کبھی کبھی<br>d. Most of the time زیادہ تر<br>e. Always ہمیشہ
13. During the past 12 months how often have you been so worried about something that you could not sleep at night?<br>گزشتہ 12 مہینے میں کتنی مرتبہ پریشانی کے باعث آپ نیند نہ کر سکے؟	a. Never کبھی نہیں<br>b. Rarely بہت کم<br>c. Sometimes کبھی کبھی<br>d. Most of the time زیادہ تر<br>e. Always ہمیشہ

Question	Options
Questions related to school grade.<br>14. What were your grades in your last promotion/ final term exam<br>گزشتہ سالانہ امتحان میں آپکے کتنے نمبر تھے؟	a. A+ grade/ outstanding<br>b. A grade / Excellent<br>c. B Grade/ Average/ good<br>d. C grade/ Fair<br>e. D Grade/ Poor/ fail

QUESTIONNAIRE
SCREEN FOR ADOLESCENT VIOLENCE EXPOSURE (SAVE)
(Questions relevant to abuse have been selected)

Question	Options	Never	Hardly Ever	Sometimes	Almost always
15.Grownups shout at me.<br>بڑے مجھ پر چیختے ہیں ؟	At home				
	At school				
16.Grownups hit me<br>بڑے مجھے مارتے ہیں ۔	At home				
	At school				
17. Someone my age hits me<br>مجھے میرے ہم عمر مارتے ہیں۔	At home				
	At school				

Questions related to generalized anxiety disorder have been selected.
Circle one option for each question.

Question	Never کبھی نہیں	Sometimes کبھی کبھی	Often زیادہ تر
18. Worry about doing better at things.<br>پریشان رہتا ہوں کہ چیزوں کو بہتر طریقے سے کر سکوں	0	1	2
19. worry about past behaviour<br>اپنے گزشتہ رویے کے بارے میں پریشان رہتا ہوں	0	1	2
20. worry about doing the wrong thing<br>غلطی کرنے کے بارے میں پریشان رہتا ہوں	0	1	2
21. worry about things in the future<br>مستقبل کے بارے میں پریشان رہتا ہوں	0	1	2
22. Afraid of making mistakes<br>غلطی کرنے سے ڈرتا ہوں	0	1	2
23. overly anxious to please people<br>پریشان رہتا ہوں کہ لوگوں کو خوش رکھ سکوں	0	1	2
Total =5			

MOTHER FORM

This questionnaire lists various attitudes and behaviours of parents. As you remember your MOTHER in your first 16 years would you place a tick in the most appropriate box next to each question

	Very like (بالکل ایسی)	Moderately like (درمیانہ ایسی)	Moderately unlike (درمیانہ نہیں)	Very unlike (بالکل نہیں)
1. Spoke to me in a warm and friendly voice<br>مجھ سے دوستی اور گرمجوشی سے بات کرتی تھیں	☐	☐	☐	☐
2. Did not help me as much as I needed<br>میری مدد اتنی نہیں کرتی جتنی مجھے ضرورت ہے	☐	☐	☐	☐
3. Let me do those things I liked doing<br>مجھے میری مرضی کے کام کرنے دیتی ہے	☐	☐	☐	☐
4. Seemed emotionally cold to me<br>میرے ساتھ سرد رویہ اختیار کرتی ہے	☐	☐	☐	☐
5. Appeared to understand my problems and worries<br>میرے مسائل اور پریشانیاں سمجھتی ہے	☐	☐	☐	☐
6. Was affectionate to me<br>مجھ سے پیار کرتی ہیں	☐	☐	☐	☐
7. Liked me to make my own decisions<br>چاہتی ہیں کہ میں اپنے فیصلے خود کروں	☐	☐	☐	☐
8. Did not want me to grow up<br>وہ نہیں چاہتی ہیں کہ میں بڑوں جیسا بن سکوں	☐	☐	☐	☐
9. Tried to control everything I did<br>وہ سب کچھ کنٹرول کرنے کی کوشش کرتی ہیں	☐	☐	☐	☐
10. Invaded my privacy<br>وہ میری پرائیویسی کا خیال نہیں کرتیں	☐	☐	☐	☐
11. Enjoyed talking things over with me<br>وہ میرے ساتھ بات کر کے لطف اندوز ہوتی ہیں	☐	☐	☐	☐
12. Frequently smiled at me<br>وہ اکثر میری طرف مسکراتی ہیں	☐	☐	☐	☐
13. Tended to baby me<br>مجھے لاڈ کرتی ہیں	☐	☐	☐	☐
14. Did not seem to understand what I needed or wanted<br>میری ضرورت اور خواہش کو نہیں سمجھ سکتی ہیں۔	☐	☐	☐	☐
15. Let me decide things for myself<br>مجھے اپنی چیزوں کا فیصلہ کرنے دیتی ہیں	☐	☐	☐	☐
16. Made me feel I wasn't wanted<br>مجھے محسوس کرائیں ہیں کہ میں ان کا پسندیدہ نہیں ہوں	☐	☐	☐	☐
17. Could make me feel better when I was upset<br>ناراض ہونے پر مجھے منا [illegible] سکتی	☐	☐	☐	☐
18. Did not talk with me very much<br>مجھ سے زیادہ باتیں نہیں کرتی	☐	☐	☐	☐
19. Tried to make me feel dependent on her/him<br>مجھے محسوس کراتی کہ میں ان پر انحصار کرتا ہوں/کرتی ہوں	☐	☐	☐	☐
20. Felt I could not look after myself unless she/he was around<br>سمجھی کہ میں ان کے بغیر اپنی دیکھ بھال نہیں کر سکتا/سکتی	☐	☐	☐	☐
21. Gave me as much freedom as I wanted<br>مجھے میری ضرورت کے مطابق آزادی دیتی	☐	☐	☐	☐
22. Let me go out as often as I wanted<br>مجھے جتنا میں چاہتا اتنا باہر جانے کی اجازت دیتی	☐	☐	☐	☐
23. Was overprotective of me<br>مجھ پر حد سے زیادہ حفاظتی رویہ رکھتی	☐	☐	☐	☐
24. Did not praise me<br>میری تعریف نہیں کرتی	☐	☐	☐	☐
25. Let me dress in any way I pleased<br>مجھے میری مرضی کے کپڑے پہننے دیتی ہے	☐	☐	☐	☐

[illegible] may be freely downloaded and distributed on condition no change is made to the content. The information in this document is not intended as a substitute for [illegible] diagnosis or treatment. Not to be used for commercial purposes and not to be hosted electronically outside of the Black Dog Institute website

FATHER FORM

This questionnaire lists various attitudes and behaviours of parents. As you remember your FATHER [illegible] years would you place a tick in the most appropriate box next to each question ہاں ، اوسطاً ہاں ، اوسطاً نہیں ، بالکل نہیں

	Very like	Moderately like	Moderately unlike	Very unlike
1 Spoke to me in a warm and friendly voice مجھ سے دوستی اور گرم جوشی سے بات کرتے				
2 Did not help me as much as I needed میری مدد اتنی نہیں کرتے جتنی مجھے ضرورت ہے				
3 Let me do those things I liked doing مجھے میری مرضی سے کام کرنے دیتے				
4 Seemed emotionally cold to me میرے ساتھ سرد رویہ اختیار کرتے				
5 Appeared to understand my problems and worries میرے مسائل اور پریشانیاں سمجھتے ہیں				
6 Was affectionate to me مجھ سے پیار کرتے ہیں				
7 Liked me to make my own decisions چاہتے ہیں کہ میں اپنے فیصلے خود کروں				
8 Did not want me to grow up وہ نہیں چاہتے کہ میں بڑوں جیسا بن سکوں				
9 Tried to control everything I did میرے سارے کام کنٹرول کرنے کی کوشش کرتے ہیں				
10 Invaded my privacy وہ میری پرائیویسی کا خیال نہیں کرتے				
11 Enjoyed talking things over with me وہ میرے ساتھ بات کرتے لطف اندوز ہوتے ہیں				
12 Frequently smiled at me وہ اکثر میری طرف مسکراتے ہیں				
13 Tended to baby me مجھ سے لاڈ کرتے ہیں				
14 Did not seem to understand what I needed or wanted میری ضروریات اور خواہشوں کو نہیں سمجھ سکتے				
15 Let me decide things for myself مجھے اپنی چیزوں کا فیصلہ کرنے دیتے ہیں				
16 Made me feel I wasn't wanted مجھے احساس دلاتے ہیں کہ میں ان کا پسندیدہ نہیں ہوں				
17 Could make me feel better when I was upset ناراضی ہونے پر مجھے منا سکتے				
18 Did not talk with me very much مجھ سے زیادہ باتیں نہیں کرتے				
19 Tried to make me feel dependent of her/him مجھے احساس دلاتے ہیں کہ میں ان پر انحصار کرتا / کرتی ہوں				
20 Felt I could not look after myself unless she/he was around سمجھتے ہیں کہ میں ان کے بغیر اپنا / اپنی دیکھ بھال نہیں کر سکتا / سکتی				
21 Gave me as much freedom as I wanted مجھے میری مرضی کے مطابق آزادی دیتے ہیں				
22 Let me go out as often as I wanted مجھے جتنا میں چاہتا / چاہتی اتنا باہر جانے کی اجازت دیتے				
23 Was overprotective of me مجھ پر حد سے زیادہ حفاظتی رویہ رکھتے				
24 Did not praise me میری تعریف نہیں کرتے				
25 Let me dress in any way I pleased مجھے میری مرضی کے کپڑے پہننے دیتے ہیں				

[illegible] be freely downloaded and distributed on condition no change is made to the content. The information in this document is [illegible] diagnosis or treatment. Not to be used for commercial purposes and not to be hosted electronically outside of the Black Dog Institute's website.
